# Cardiology in numbers: coronary artery disease in the Netherlands

**DOI:** 10.1007/s12471-026-02058-2

**Published:** 2026-07-09

**Authors:** Maaike M. Roefs, Giovanni Amoroso, Cyril Camaro, Edgar J. Daeter, Maik J. Grundeken, Arnoud W. J. van ‘t Hof, Robert A. F. de Lind van Wijngaarden, Erik Lipsic, Martijn Meuwissen, Pim van der Harst, Dennis van Veghel

**Affiliations:** 1 Netherlands Heart Registration, Utrecht, The Netherlands; 2 Department of Cardiology, OLVG, Amsterdam, The Netherlands; 3 Department of Cardiology, Radboud University Medical Center, Nijmegen, The Netherlands; 4 Department of Cardiac Surgery, St Antonius Hospital, Nieuwegein, The Netherlands; 5 Department of Cardiology, Amsterdam University Medical Centers, Amsterdam, The Netherlands; 6 Department of Cardiology, Maastricht University Medical Center, Maastricht, The Netherlands; 7 Department of Cardiothoracic Surgery, Amsterdam University Medical Centers, Amsterdam, The Netherlands; 8 Department of Cardiology, University Medical Center Groningen, Groniningen, The Netherlands; 9 Department of Cardiology, Amphia Hospital, Breda, The Netherlands; 10 Netherlands Heart Registration, Utrecht, The Netherlands

## Introduction

Coronary artery disease (CAD), commonly captured in global burden estimates as ischemic heart disease (IHD), remains the leading cause of death worldwide, with an estimated number of 8,990,000 (95% confidence interval: 8,290,000–9,550,000) deaths globally in 2021 [1]. Within Europe, the Netherlands ranks among the countries with the lowest age-standardized mortality for both cardiovascular disease and ischemic heart disease [2]. Despite this, CAD remains one of the top three contributors to disease burden in the Netherlands, as measured by Disability Adjusted Life Years (DALYs), with 9.54 DALYs per 1000 people in 2022 [3]. The absolute number of deaths attributed to CAD has steadily declined in the Netherlands, from 24,536 deaths in 1980 to 7,919 deaths in 2024. Of these deaths in 2024, 4,894 (61,8%) occurred in men and 3,026 (38,2%) in women [4].

## Key facts and figures

In 2024, there were 54,806 hospital admissions for CAD in the Netherlands. Among these admissions, 38,141 involved men (69.6%) and 16,665 involved women (30.4%), indicating a clear male predominance. The average length of hospital stay was 4 days for both men and women [4]. In addition, CAD ranks among the top 10 most expensive conditions in the Netherlands, with estimated healthcare expenditure of 1439 million euros in 2019, accounting for 21% of cardiovascular diseases-related expenditures and 1.5% of total healthcare expenditure [3].

Treatment options for CAD include medical treatment and revascularization, either by coronary artery bypass grafting (CABG) or percutaneous coronary intervention (PCI). Information on these revascularization procedures is collected within the Netherlands Heart Registration (NHR), a nation-wide quality registry for cardiac interventions that includes data on patient characteristics, intervention variables, and outcomes [5]. Between 2013 and 2025, a total of 90,580 isolated CABGs were performed in the Netherlands, corresponding to an annual average of 6,967 procedures (Tab. 1). The annual number of procedures decreased by 17% over this period, from 7,929 in 2013 to 6,620 in 2025. Until 2016, most isolated CABGs were performed electively; for example 4604 procedures (59.4%) were elective in 2014. In recent years, however, the number of elective procedures has declined, with only 2,567 elective cases in 2025, representing 38,8% of all isolated CABG procedures. Data from 2024 show a marked male predominance among patients undergoing CABG, with 82.1% of all procedures performed in men [6].

**Table 1:** Number of revascularization interventions for coronary artery disease per year in the Netherlands

	2013	2014	2015	2016	2017	2018	2019	2020	2021	2022	2023	2024	2025
Isolated CABG	7,929	7,754	7,538	7,289	7,255	6,898	7,384	6,541	6,180	6,330	6,392	6,470	6,620
*Elective*	*–*	*4,604*	* 3,948*	* 3,475*	* 3,339*	* 2,905*	* 3,211*	* 2,598*	* 2,340*	* 2,615*	* 2,475*	* 2,529*	* 2,567*
*Urgent/emergency/salvage*	*–*	*3,147*	* 3,588*	* 3,814*	* 3,913*	* 3,699*	* 4,171*	* 3,936*	* 3,825*	* 3,711*	* 3,912*	* 3,935*	* 4,049*
PCI	–	–	40,154	39,835	40,279	40,128	40,701	37,714	38,794	37,663	37,137	37,476	36,875
*Elective*	–	–	–	–	–	*16,850*	*17,051*	*14,387*	*15,366*	*15,150*	*14,577*	*15,058*	*13,815*
*Non-STEMI*	–	–	–	–	–	*12,556*	*13,178*	*12,640*	*12,447*	*11,681*	*11,765*	*11,525*	*10,603*
*STEMI*	–	–	–	–	–	*10,579*	*10,427*	*10,528*	*10,845*	*10,769*	*10,668*	*10,730*	* 9,991*
**Total revascularization**	**–**	**–**	**47,692**	**47,124**	**47,534**	**47,026**	**48,085**	**44,255**	**44,974**	**43,993**	**43,529**	**43,946**	**43,495**

Subrows may not sum to the reported total because some interventions had missing information on indication.

Abbreviations: *CABG* Coronary Artery Bypass Graft, *PCI* Percutaneous Coronary Intervention, *STEMI* ST-Segment Elevation Myocardial Infarction

Between 2015 and 2025, a total number of 426,757 PCIs were performed in the Netherlands, corresponding to an annual average of 38,796 procedures (Tab. 1). Over this 11-year period, the annual number of PCIs remained relatively stable during the first 5 years but declined from 2020 onward. Based on the data from 2018 onward, elective PCIs were less common than non-elective procedures (in case of non-STEMI or STEMI). In 2024, 15,058 PCIs (40.2%) were elective, 11,525 (30.8%) were performed for non-STEMI and 10,730 (28.6%) for STEMI. Similar to CABG, PCI patients were predominantly male, with men accounting for 73.2% of all patients undergoing PCI in 2024 [6].

## Conclusion

CAD is a common cardiovascular condition, worldwide and in the Netherlands. Although the number of CABG and PCI procedures has gradually declined in recent years, CAD continues to have a substantial impact on major life events and healthcare utilization.

## Supplementary Information

Full list of collaborators.

## Data Availability

All data supporting the findings of this work are included in the article.
