# Supplementary material for: Cardiology in numbers: coronary artery disease in the Netherlands
Source: Neth Heart J. 2026 Jul 9;34(7-8):283–4. doi: 10.1007/s12471-026-02058-2 (PMC13376289; doi:10.1007/s12471-026-02058-2)
Supplement: Supplementary file 1 — Full list of collaborators. [file 12471_2026_2058_MOESM1_ESM.docx]

**Cardiothoracic Surgery Registration Committee**

| **Name** | **Hospital** |
| --- | --- |
| Mr. E.J. Daeter | St. Antonius Hospital |
| Dr. S. Bramer | Amphia Hospital |
| Dr. R.A.F. de Lind van Wijngaarden | Amsterdam University Medical Centres |
| Mr. B.M.J.A. Koene | Catharina Hospital |
| Mr. D. Stecher | Frisius Medical Centre |
| Dr. G.J.F. Hoohenkerk | Haga Hospital |
| Dr. A.L.P. Markou | Isala Hospital |
| Mr. A. de Weger | Leiden University Medical Centre |
| Dr. P. Segers | Maastricht University Medical Centre+ |
| Dr. R.G.H. Speekenbrink | Medical Spectrum Twente |
| Mr. V.G. Hindori | OLVG Amsterdam |
| Mr. H. van Wetten | Radboud University Medical Centre |
| Dr. M.M. Mokhles | Utrecht University Medical Centre |
| Ms. I.J. Wijdh – den Hamer | University Medical Centre Groningen |

**PCI Registration Committee**

| **Name** | **Hospital** |
| --- | --- |
| Dr. M. Meuwissen | Amphia Hospital |
| Dr. J. Cheng | Albert Schweitzer Hospital |
| Dr. M. Grundeken | Amsterdam University Medical Centres |
| Dr. K. Teeuwen | Catharina Hospital |
| Dr. S. Hubbers | Elisabeth-TweeSteden Hospital (ETZ) |
| Dr. R. Diletti | Erasmus Medical Centre |
| Dr. J. Brouwer | Frisius Medical Centre |
| Dr. B.J. Sorgdrager | Haaglanden Medical Centre |
| Mr. C.E. Schotborgh | Haga Hospital |
| Dr. T. Meijers | Isala Hospital |
| Dr. J. Polad | Jeroen Bosch Hospital |
| Dr. R. Scherptong | Leiden University Medical Centre |
| Dr. E. Bakker | Maasstad Hospital |
| Prof. Dr. A.J.W. van 't Hof | Maastricht University Medical Centre+ |
| Mr. F. Spano | Meander Medical Centre |
| Mr. K.G. van Houwelingen | Medical Spectrum Twente |
| Dr. M. Ewing | Northwest Hospital Group |
| Dr. G. Amoroso | OLVG Amsterdam |
| Mr. C. Camaro | Radboud University Medical Centre |
| Dr. P.W. Danse | Rijnstate Hospital |
| Dr. K. Sjauw | St. Antonius Hospital |
| Dr. R. van Bommel | Tergooi Medical Centre |
| Dr. W.T. Ruifrok | Treant Care Group |
| Dr. A. Kraaijeveld | Utrecht University Medical Centre |
| Dr. E. Lipsic | University Medical Centre Groningen |
| Dr. L. Hoebers | VieCuri Medical Centre |
| Mr. R. Erdem | ZorgSaam Hospital |
| Dr. L. Ruiters | Zuyderland Medical Centre |
